# Supplementary material for: Differential Effect of Non-Thermal Plasma RONS on Two Human Leukemic Cell Populations
Source: Cancers (Basel). 2021 May 18;13(10):2437. doi: 10.3390/cancers13102437 (PMC8157554; doi:10.3390/cancers13102437)
Supplement: Supplementary file 1 [file cancers-13-02437-s001.zip › cancers-1185365-supplementary.pdf]

cancers

# Supplementary Materials: Differential Effect of Non-Thermal Plasma RONS on Two Human Leukemic Cell Populations

**Hager Mohamed, Eric Gebski, Rufranshell Reyes, Samuel Beane, Brian Wigdahl, Fred C. Krebs, Katharina Stapelmann and Vandana Miller**

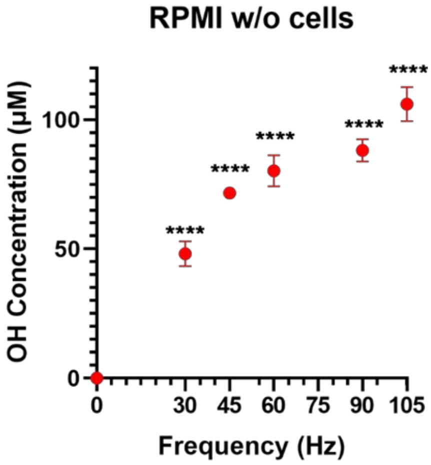


**Figure S1.** NTP-exposure of RPMI results in a frequency-dependent increase in OH immediately (T0) following exposure. Data are presented as mean ± SD from one experiment ($n$ = 3). Significance was calculated using an unpaired Student's t-test and determined with respect to the mock-exposed (0 Hz) control (**** $p < 0.0001$).

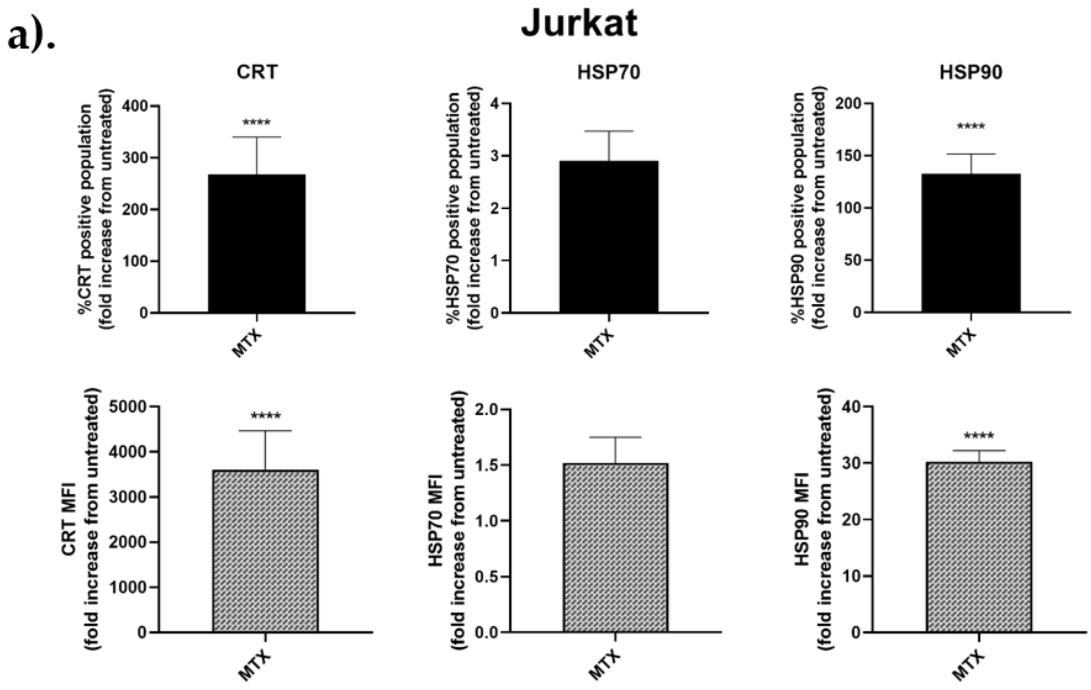

b).

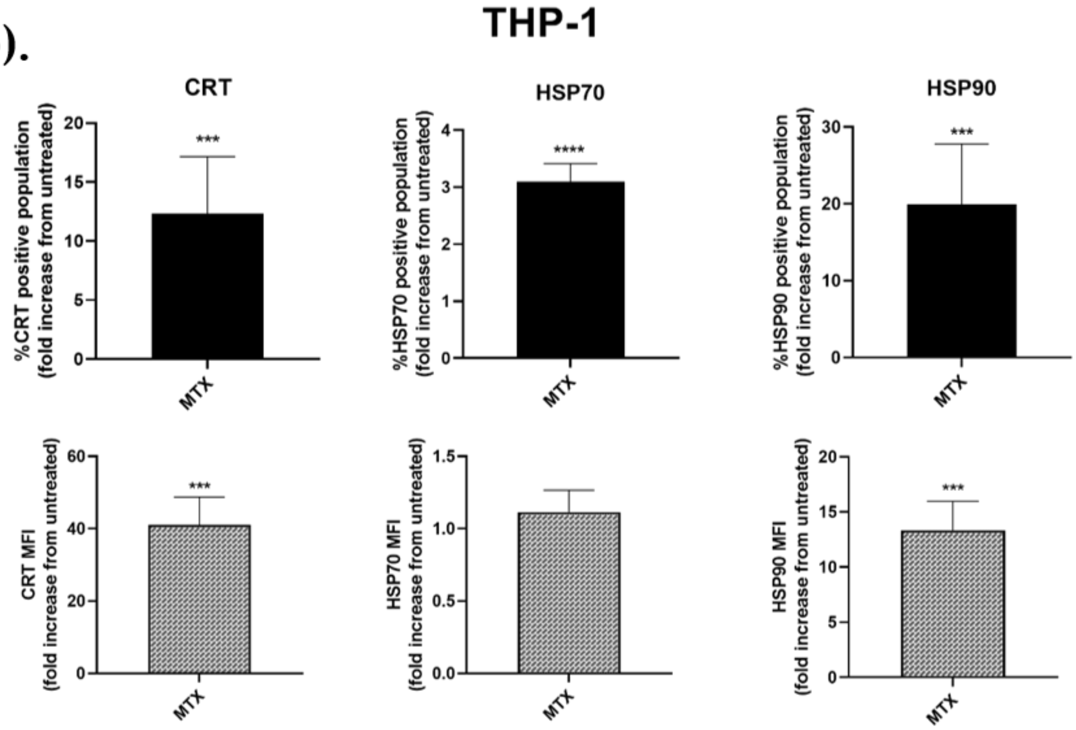


**Figure S2.** Treatment of Jurkat or THP-1 cells with MTX results in robust increase in the display of pro-phagocytic markers. Display of CRT, HSP70, and/or HSP90 is increased 24 h following treatment with the chemotherapeutic drug mitoxantrone (MTX) of (**a**) Jurkat and (**b**) THP-1 cells. Data are presented as mean ± SEM. Significance was calculated using a Kruskal-Wallis test with Dunnett's post-hoc test (*** $p < 0.001$, **** $p < 0.0001$).
